# Physiological and Molecular Response Modifications by Ultraviolet-C Radiation in *Plutella xylostella* and Its Compatibility with *Cordyceps fumosorosea*

**DOI:** 10.3390/ijms23179800

**Published:** 2022-08-29

**Authors:** Muhammad Musa Khan, Ze-Yun Fan, Irfan Ali Sabir, Muhammad Hafeez, Sang Wen, Jian-Hui Wu, Bao-Li Qiu

**Affiliations:** 1 Chongqing Key Laboratory of Vector Insects, College of Life Sciences, Chongqing Normal University, Chongqing 401331, China; 2 Key Laboratory of Bio-Pesticide Innovation and Application of Guangdong Province, South China Agricultural University, Guangzhou 510640, China; 3 School of Agriculture and Biology, Shanghai Jiao Tong University, Shanghai 200240, China; 4 State Key Laboratory Breeding Base for Zhejiang Sustainable Pest and Disease Control, Institute of Plant Protection and Microbiology, Zhejiang Academy of Agricultural Sciences, Hangzhou 310021, China

**Keywords:** UV-C radiation, virulence, antioxidant enzyme, detoxification enzyme, qRT-PCR, entomopathogenic fungi

## Abstract

Ultraviolet-C (UV-C) radiation significantly impacts living organisms. UV-C radiation can also be used as a pest management tool. Therefore, this study was designed to investigate the effect of UV-C radiation on the physiology and gene expression level of *Plutella xylostella*, a destructive vegetable pest. Results showed that, after exposure to UV-C radiation for 3, 6, 12, and 24 h, the activity of SOD (superoxide dismutase) and CAT (catalase) of *P. xylostella* increased, while the activity of PPO (polyphenol oxidase), POD (peroxidase), AChE (acetylcholinesterase), CarE (carboxylesterase), and ACP (acid phosphatase) decreased with increased exposure time. Correlation coefficient analyses indicated that the activity of CAT correlated positively, while PPO and CarE correlated negatively, with exposure time. Gene regulation analysis via qRT-PCR confirmed a significant increase in regulation in *CAT*, *CarE*, and *PPO*-related genes. We also investigated the effect of UV-C exposure on the virulence of *Cordyceps fumosorosea* against *P. xylostella*. Here, results indicated that when the fungal treatment was applied to larvae before UV-C radiation, the virulence of *C. fumosorosea* was significantly reduced. However, this decline in virulence of *C. fumosorosea* due to UV-C exposure remained only for one generation, and no effect was observed on secondary infection. On the other hand, when larvae were exposed to UV-C radiation before fungal application, the mortality rate significantly increased as the exposure time to UV-C radiation increased. From the current study, it could be concluded that UV-C exposure suppressed the immunity to *P. xylostella,* which later enhanced the virulence of entomopathogenic fungi. Moreover, the study also suggested that UV irradiation is an effective pest management tool that could be incorporated into pest management strategies, which could help reduce pesticide application, be economically beneficial for the farmer, and be environmentally safe.

## 1. Introduction

Stress factors, both abiotic and biotic, play important roles in the development and growth of an insect. Environmental stresses such as ozone exposure, ultraviolet irradiation, and temperature stress significantly impact insects. For many organisms, ultraviolet (UV) radiation is a significant source of stress [1]. UV irradiation is an important ecological stressor for biological entities [2]. Ultraviolet-C longwave (UV-C) is a wavelength of electromagnetic radiation between 100 and 280 nm. Most moths can tolerate UV irradiation; however, blacklight, a synthetic form of UV-light, has been extensively used to control nocturnal moths [3,4]. Previous studies reported a direct effect of UV light on insect behavior [5], development, biology, and physiology [6,7], and biochemistry [8], but there is no study available reporting direct mortality of moths caused by UV light.

Insects and microorganisms are reported to have detrimental effects from UV-C radiation exposure [9,10,11]. *Tribolium castaneum* (Herbst, 1797), *Tribolium confusum* (DuVal, 1863), *Cadra cautella* (Walker, 1863), and *Trogoderma granarium* (Everts, 1899) are a few grain storage pests that have been examined for the use of UV-C radiation to manage them [11]. The silkworm *Bombyx mori* (Linnaeus, 1758) larvae are particularly reported to be vulnerable to the lethal effects of UV-C radiation [12]. Studies have reported that exposure to UV-C radiation could kill nymphs of *Periplaneta americana* (Linnaeus, 1785) and other pests [13,14]. UV-light exposure affects insect biology and is also known to negatively influence the virulence of entomopathogenic fungi [15], which may provide an alternative to synthetic insecticidal control. Most fungi can be killed by direct solar radiation exposure because UV radiation is the most dangerous and mutagenic light form of the solar spectrum [16].

Under normal conditions, antioxidants are stable within insects and help maintain regular metabolic activity. However, UV light can cause oxidative stress and damage insect protein functional activity [17,18]. Recent studies have proposed that UV light can significantly change antioxidant enzyme activity [19,20,21,22] and detoxification enzyme activity [21]. In addition, UV light exerts strong genotoxic effects via DNA damage, induces mutations, and could also be carcinogenic [23]. Several studies [24,25,26,27] reported that enzyme activity in any organism depends on gene regulation. Therefore, small changes in gene expression may have a significant impact on enzyme activity. As UV-C radiation can cause gene mutation, it is also possible that variation in enzymatic activity could result from changes in gene expression.

The diamondback moth, *Plutella xylostella* (Linnaeus, 1758) (Lepidoptera: Plutellidae), is the most important cruciferous vegetable pest in the world [28]. Due to short generation time, high fecundity, and indiscriminate use of insecticides, *P. xylostella* has become resistant to 81 insecticides to date [29,30], including spinosad [31,32], avermectins (abamectin and emamectin benzoate) [33], indoxacarb [34] and *Bacillus thuringiensis* [31,35]. High insecticide resistance development in *P. xylostella* made it a difficult pest to manage [36,37,38]. Therefore, it is necessary to promote alternative strategies such as entomopathogenic fungi to control *P. xylostella* and minimize synthetic insecticides [39].

In the 1990s, *Cordyceps fumosorosea* (Wize, 1904) (formerly known as *Isaria fumosorosea* Wize) [40] was reportedly the most common entomopathogenic fungi in Asia, Russia, the USA, South America, and India. Under favorable conditions, *C. fumosorosea* can cause a significant reduction in insect pest populations [41]. Apart from its efficacy and low cost of production, the use of *C. fumosorosea* has other advantages, including broader insecticidal activity, a diversified host range, and safety for humans and other non−target organisms [42]. *C. fumosorosea* is a highly effective antagonist of *P. xylostella* [43,44,45]. Several studies have confirmed the potential of *C. fumosorosea*, such as *Bemisia tabaci* (Gennadius) [46,47], *Empoasca vitis* (Gothe, 1875) [48], *Aphis gossypii* (Glover) [49] and *Corythucha ciliata* (Say) [50]. The corpses of host insects loaded with conidia generate cadavers and play an important role in the secondary spread of infection [51]. Furthermore, *C. fumosorosea* is commercially available worldwide [52].

The current study was designed to test the following hypothesis: (1) UV-C radiation affects the activity of the following antioxidant enzymes: Superoxide dismutase (SOD), catalase (CAT), peroxidase (POD), polyphenol oxidase (PPO), and glutathione S-transferase (GST) and the following detoxifying enzymes: Acetylcholinesterase (AChE), carboxylesterase (CarE), alkaline phosphatase (ALP) and acidic phosphatase (ACP); (2) The alteration in enzyme activity is due to alteration in gene expression; (3) The alteration in physiology will affect the virulence of *C. fumosorosea*; (4) UV-C exposure to the *P. xylostella* is effective before or after the application of *C. fumosorosea*.

## 2. Results

### 2.1. Effect of UV-C Radiation on the Physiology of Plutella xylostella

#### 2.1.1. Antioxidant Enzyme Activities

**Figure 1:**
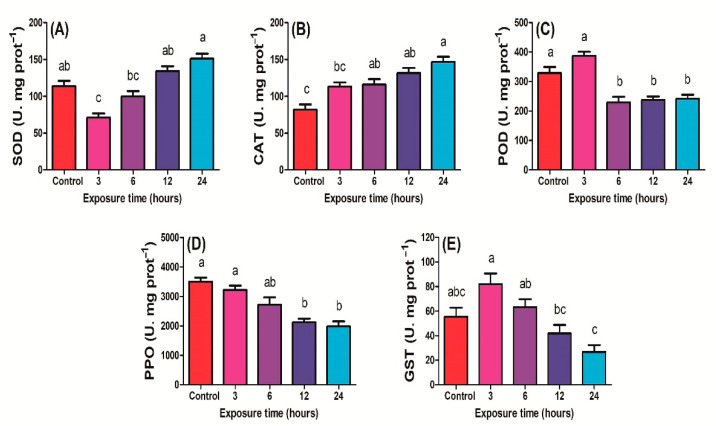
Activities of different antioxidant enzymes (**A**) SOD, (**B**) CAT, (**C**) POD, (**D**) PPO, and (**E**) GST of *Plutella xylostella* larvae exposed to UV−light for control (0 h), 3, 6, 12, and 24 h. The bars indicate the mean values of four replications. Standard error bars indicate the standard deviation of the mean. Lowercase letters indicate the significant difference among the treatments at *P* < 0.05. Similar letters have no significant difference among treatments.

The *P. xylostella* larvae were exposed to UV-C irradiation for 3 h, 6 h, 12 h, and 24 h, and the activity of enzymes was measured. SOD activity was significantly lower after 3 h of exposure to UV-C radiation compared to the control, 12 h, and 24 h, while the activity of SOD in 3 and 6 h of exposure was not affected compared to each other (F_4,14_ = 13.4; *P* < 0.01) (Figure 1A). The CAT was higher in larvae exposed to UV-C radiation for 6, 12, and 24 h compared to control (F_4,14_ = 12.9; *P* < 0.01) (Figure 1B). POD activity in *P. xylostella* third instar larvae exposed to UV-C for 3 h was not changed compared to the control, while the activity was significantly lower after 6, 12, and 24 h of exposure to UV-C radiation (F_4,14_ = 18.6; *P* < 0.01) (Figure 1C). The activity of PPO was significantly lower in treatments exposed to UV-C radiation for 12 and 24 h as compared to control and to 3 h of exposure (F_4,14_ = 14.8; *P* < 0.01) (Figure 1D). The GST activity was highest when third instar larvae were exposed to UV-C for 3 h compared to the control and other treatments. As exposure time increased, a gradual decline in GST was observed (F_4,14_ = 6.64; *P* < 0.01) (Figure 1E).

#### 2.1.2. Detoxification Enzyme Activity

**Figure 2:**
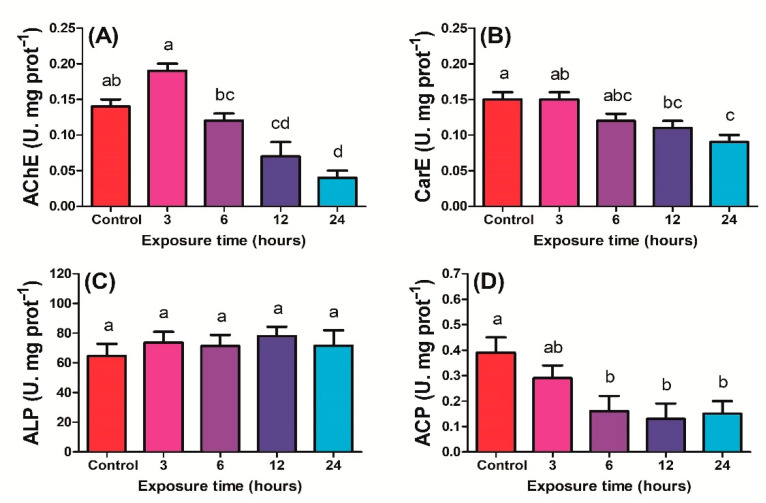
Activities of detoxification enzymes (**A**) AChE, (**B**) CarE, (**C**) ALP, and (**D**) ACP of *Plutella xylostella* larvae exposed to UV−light for control (0 h), 3, 6, 12, and 24 h. The bars indicate the mean value of four replications, and standard error bars indicate the standard deviation of the mean. Lowercase letters indicate a significant difference among the treatments at *P* < 0.05, and similar letters have no significant difference among treatments.

After 3 and 6 h of exposure to UV-C, no significant change in AChE activity of *P. xylostella* third instar larvae was observed compared to control, while 12 and 24 h activity was significantly decreased compared to control (F_4,14_ = 25.7; *P* < 0.01) (Figure 2A). The CarE activity decreased significantly when *P. xylostella* was exposed to UV-C for 12 and 24 h compared to the control, while no significant change in CarE activity was observed after control, 3 h or 6 h of exposure (F_4,14_ = 8.96; *P* < 0.01) (Figure 2B). ALP activity variations in response to exposure time were found to be non-significant (F_4,14_ = 0.36; *P* > 0.05) (Figure 2C). ACP activity was considerably higher in control compared to 6, 12 and 24 h exposed to UV-C radiation. Variation in ACP activity was non-significant when *P. xylostella* was exposed to UV-C radiation for 6, 12, and 24 h (F_4,14_ = 4.01; *P* < 0.05) (Figure 2D).

#### 2.1.3. Correlation of Enzyme Activity with UV Exposure Time

**Figure 3:**
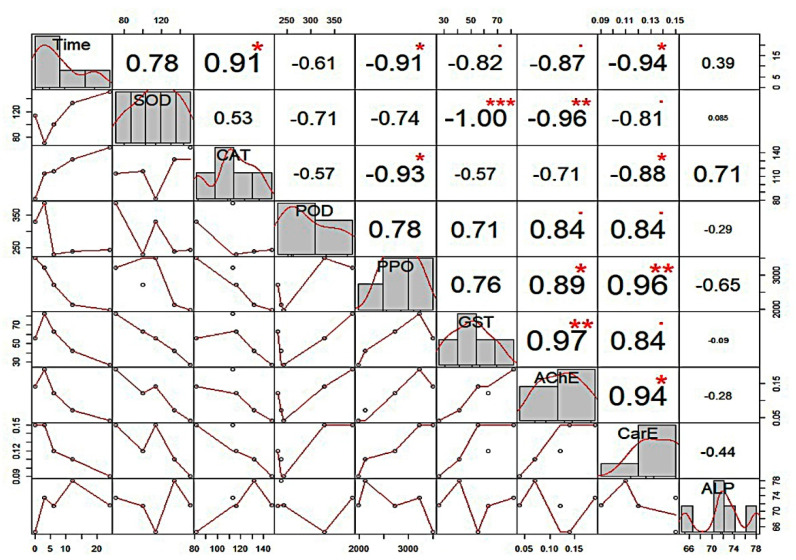
Pearson correlation matrix of UV−light exposure time and *Plutella xylostella* larvae enzyme activity. The distribution of each variable is shown on the diagonal. The bivariate scatter plots with a fitted line are displayed on the bottom of the diagonal. The correlation value and the significance level as stars are on the top of the diagonal. Each significance level is associated to a symbol: *P*-values (0, 0.001, 0.01, 0.05, 0.1) < = > symbols (“***”, “**”, “*”, “.”, “ ”).

The main objective of conducting Pearson correlation between exposure time and enzyme activity was to determine whether the enzyme has a significant correlation with time so the genes regulating enzyme could be selected to determine their gene expression. The results demonstrated that SOD and ALP had insignificant positive correlations, while POD, AChE, and ACP showed insignificant negative correlations with UV-C radiation exposure time. Alternatively, CAT correlated significantly (r = 0.905; *P* < 0.05) positively, while PPO (r = −0.908; *P* < 0.05) and CarE (r = −0.940; *P* < 0.05) correlated significantly negatively with time under UV-C radiation exposure. Correlation coefficients between exposure time and enzyme activity and between enzymes are shown in Figure 3. The results also showed the association between the activities of different enzymes. The enzymes with *P* < 0.05 were picked for gene expression analysis.

### 2.2. Effect of UV Radiation on Gene Expression of Plutella xylostella

**Figure 4:**
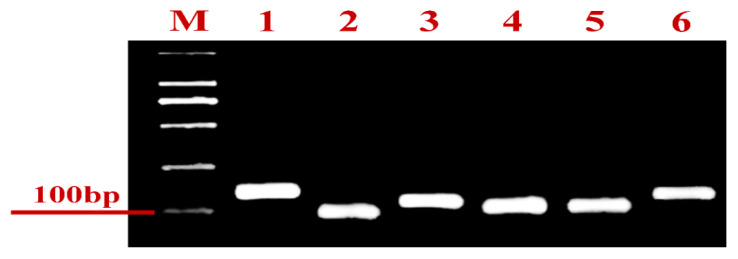
Agarose gel electrophoresis of six genes regulating enzyme of significant correlation with UV exposure. M, Molecular marker. Templates in the polymerase chain reactions (PCRs) were as follows: (1) *CAT1*; (2) *CAT2*; (3) *CarE1*; (4) *CarE2*; (5) *PPO1*; and (6) *PPO2*.

Two genes of each enzyme showing a significant correlation with UV-C exposure time (*CAT, PPO*, and *CarE*) were selected and subjected to gene expression analysis. Gene expression was analyzed after 0, 3, 6, 12, and 24 h exposure to UV-C radiation, and primer pairs were tested before gene expression analysis (Figure 4).

**Figure 5:**
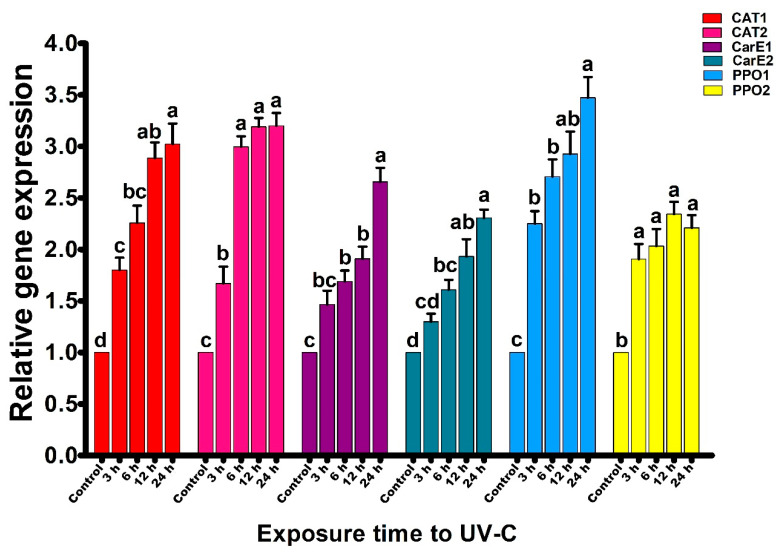
The gene regulation of Catalase-related (CAT1 and CAT2), Carboxylesterase-related (CarE1 and CarE2), and Prophenoloxidase-related (PPO1 and PPO2) genes of *Plutella xylostella* larvae after 0 (control), 3, 6, 12, and 24 h exposures to UV−light. The bars indicate the mean values of four replications; standard error bars indicate the standard deviation of the mean, and error bars indicate the mean deviation. Small letters indicate a significant difference among the treatments at *P* < 0.05, and similar letters have no significant difference among treatments.

Results showed that UV-C radiation exposure significantly increased the gene expression of *CAT1* compared with the control (Figure 5). The gene expression was 3.02-fold higher after 24 h than in control (F_4,14_ = 32.2; *P* < 0.01). Gene expression of the *CAT2* gene increased; however, gene expression had stabilized after 6, 12, and 24 h. Nonetheless, gene expression was significantly elevated in all treatments compared with the control (F_4,14_ = 85.8; *P* < 0.01). The maximum fold change was observed after 24 h of exposure to UV-C radiation (3.20-fold). Expression levels of the genes *CarE1* (F_4,14_ = 30.6; *P* < 0.01) and *CarE2* (F_4,14_ = 26.6; *P* < 0.01), which regulate carboxylesterase activity in *P. xylostella*, were increased in treatments exposed to UV-C for 6, 12 and 24 h compared to control. The gene expression of *CarE1* increased 2.66-fold, while *CarE2* increased 2.31-fold following 24 h of UV-C exposure. *PPO1* and *PPO2* genes responsible for protoporphyrinogen oxidase activity in *P. xylostella* PPO1 were also significantly increased with UV-C radiation exposure time (F_4,14_ = 33.3; *P* < 0.01) after 24 h of exposure, *PPO1* expression had increased 3.47-fold. PPO2 had increased in all UV-C radiation treatments compared to control but had no statistical difference from each other. The maximum fold change in *PPO2* was observed after 12 h of exposure (2.34-fold).

### 2.3. Effect of UV-C Radiation on the Virulence of Cordyceps fumosorosea

**Figure 6:**
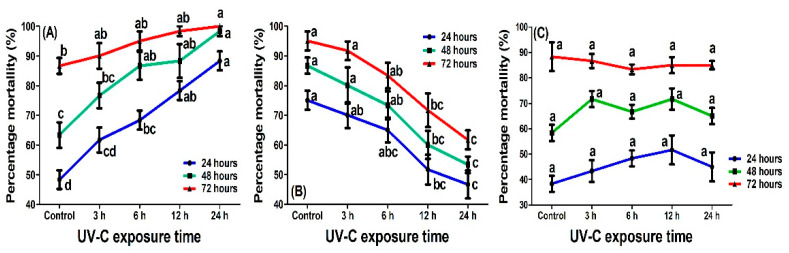
Mortality percentage of third instar larvae of *Plutella xylostella* exposed to UV-C radiation (**A**) First UV−C radiation then *Cordyceps fumosorosea* treatment; (**B**) First *C. fumosorosea* treatment then UV-C radiation; (**C**) Dead insects from (**B**) collected, F1 fungal generation regrown and virulence checked again. Each bar shows the mean value of four replications, and error bars indicate the mean deviation. Lowercase letters indicate a significant difference among the treatments at *P* < 0.05, and similar letters have no significant difference among treatments.

Virulence of *C. fumosorosea* towards *P. xylostella* third instar larvae when UV-C radiation exposure preceded the application of the conidial suspension is shown in Figure 6A. Results revealed that the virulence of *C. fumosorosea* increased. The mortalities in all UV-C-exposed treatments were non-significantly different than control, but there was a significant increase in virulence observed after 24 h (F_4,19_ = 20.2; *P* < 0.01), 48 h (F_4,19_ = 9.31; *P* < 0.01) and 72 h (F_4,19_ = 4.04; *P* < 0.01) due to 24 h of exposure to UV-C radiation.

The mortality of *P. xylostella* third instar larvae, first exposed to *C. fumosorosea* and then to UV-C, is shown in Figure 6B. Results indicated that exposure to UV-C radiation reduced the virulence of *C. fumosorosea* against *P. xylostella.* As the exposure time increased, the virulence of *C. fumosorosea* significantly decreased. There was a significant decrease of virulence observed when *C. fumosorosea*-treated *P. xylostella* larvae were exposed to UV-C radiation for 24 h (F_4,19_ = 7.8; *P* < 0.01), 48 h (F_4,19_ = 9.92; *P* < 0.01) and 72 h (F_4,19_ = 11.90; *P* < 0.01).

The effect of UV-C in the F1 generation of *C. fumosorosea* was also assessed by collecting dead larvae and isolating *C. fumosorosea* from the third instar larvae of *P. xylostella*, which were firstly treated with *C. fumosorosea* and then exposed to UV-C radiation. The results are shown in Figure 6C. There was no significant difference in the mortalities of *P. xylostella* third instar larvae in F1 generation fungi, in which the F0 generation was exposed to UV-C radiation fungi or not (control). Mortality rates among *P. xylostella* third instar larvae was non-significant after 24 h (F_4,19_ = 1.23; *P* > 0.05), 48 h (F_4,19_ = 2.75; *P* > 0.05) or 72 h (F_4,19_ = 0.32; *P* > 0.86).

## 3. Discussion

Antioxidant enzymes are an important component of the insect immune system, protecting cellular networks from oxidative damage caused by xenobiotics [53]. SOD is an essential antioxidant protein used to alleviate superoxide radicals present in cells. In the present study, SOD activity increased with increasing exposure time, suggesting that UV-light irradiation induced the formation of superoxide radicals in *P. xylostella* larvae, which has been observed previously in the Antarctic midge *Belgica antarctica* and *Dendrolimus tabulaeformis* (Tsai & Liu, 1962) [21,54]. When insects were exposed to UV-C radiation for 24 h, SOD activity increased substantially, implying that SOD was stimulated to forage for superoxide radicals and protect larvae from UV stress. However, a significant decrease was found at 3 h, followed by increased SOD activity and UV-C radiation. These results contradict the previous findings that high doses of UV irradiation suppressed protective enzyme activities in cells [55,56].

The current study demonstrated that CAT enzyme activity in *P. xylostella* third instar larvae significantly increased with increasing exposure time. Additionally, several previous studies also found that UV exposure increased CAT activity. For example, Zhou et al. [22] reported that UV-light stress significantly increased CAT activity in *Sitobion avenae* (Fabricius, 1775). Moreover, Wang et al. [21] reported that CAT activity correlated positively with UV exposure time in *D. tabulaeformis* (Tsai & Liu, 1962) females. These results support the findings of the current study.

When third instar larvae of *P. xylostella* were exposed to UV-C radiation for 3 h, a substantial increase in POD activity was observed. However, previous studies showed that oxidative stress or excess substrate negatively affected POD enzyme activity [57]. Conversely, PPO activity decreased with increasing UV exposure time in the present study. Previous studies reported that PPO catalyzes the breakdown of phenolic compounds in organisms. UV exposure-induced suppression of PPO activity has resulted in elevated phenolic compound concentration, which hinders normal cell functions [58,59].

GST is a primary antioxidant enzyme that metabolizes lipid peroxides [60]. In the current study, following 3 h of exposure, a sudden spike was observed in GST activity and declined with increasing exposure time. Meng et al. [61] reported that UV-light exposure significantly reduced GST activity. Thus, *P. xylostella* exposed to UV-C radiation appeared capable of removing lipid peroxidation products generated during UV-C radiation stress, suggesting a protective effect from elevated GST levels.

AChE, a common detoxification enzyme, plays a critical role in regulating the normal transmission of nerve impulses along synapses and preserves normal physiological functioning in different organisms [62]. AChE is commonly believed to be the target of organophosphorus and carbamate pesticides, and changes to its amino acid sequence are likely to cause insecticide resistance [63]. Additionally, AChE plays a vital role in insect growth and development. Previous studies showed that the ploidy of *Helicoverpa armigera* (Hubner) was reduced significantly with gene silencing of AChE [64]. In our study, the AChE activities in the third-instar larvae of *P. xylostella* were reduced after 24 h of UV-C radiation. CarE in *P. xylostella* showed the same proclivity as AChE, while ALP activity with exposure time was not changed. We assume that UV-C radiation can deter the transmission of neurotransmitters in insects. ALP and ACP belong to nonspecific phosphohydrolases involved in phosphate group transmission and metabolism. For ALP, the optimum pH is >7 and ACP is <7 [65]. These attributes also concluded that UV-C radiation exposure increased ROS (reactive oxygen species) production and distorted the equilibrium between acid and base within *P. xylostella*.

Previous studies [24,25,26,27] reported that genes regulate enzyme activities in organisms. Thus, small changes in gene expression may have a significant impact on enzyme activity. This appears to be the case in the current study, as the enzymes with activity levels most correlated with exposure time showed significantly altered gene expression.

*Cordyceps fumosorosea* is an important entomopathogenic fungus used to manage various insect pests [66,67,68]. Previous studies reported that *C. fumosorosea* is an effective biological control agent against *P. xylostella* as measured by affected survival, fecundity, enzyme activity, and gene expression of *P. xylostella* [69,70,71]. The current study evaluates the virulence of *C. fumosorosea* against *P. xylostella* before and after exposure to UV-C radiation. Results revealed that the virulence of *C. fumosorosea* was low if insects were exposed to UV light after treatment with conidial suspension, which may be due to damage inflicted on conidia by UV-C exposure. Indeed, several studies have shown that exposure to UV-C radiation could inhibit fungal virulence and cause molecular and physiological changes in fungal conidia [15,16,72,73]. The current study reported that exposure to UV-C radiation reduces the immunity of *P. xylostella,* which increase the effectiveness of *C. fumosorosea*. Khan et al. [7] also reported that if *Bemisia tabaci* was exposed to UV-A before application of *C. fumosorosea*, the LC_50_ decreases with an increase in exposure time, which implies that the B. tabaci become less and less immune against fungal infection due to UV-A exposure.

Many natural abiotic factors are known to limit the ability of a fungal agent to control pests biologically, but solar ultraviolet (UV) radiation (UV-A and UV-B) is probably the most detrimental environmental factor affecting the viability of fungi applied for pest control [74,75]. Most UV-tolerant isolates can survive a few hours of direct exposure to solar UV radiation, but UV-susceptible isolates succumb. In addition, the exposure of fungi to UV-B [76,77,78,79] or UV-A [80,81] may delay conidial germination of survivors and reduce fungal development, which decreases the persistence and efficacy of infective propagules in the field [82,83].

## 4. Materials and Methods

### 4.1. Plutella xylostella Rearing

*Plutella xylostella* larvae were collected from the Engineering Research Center of Biological Control at South China Agricultural University (SCAU) and reared on *Brassica rapa* L. (Chinese cabbage). Third instar larvae (emerged < 24 h) were used in all experiments. Adults were reared on a 10% honey solution in an iron-framed, plastic sieve cage (60 × 60 × 60 cm). A piece of cotton was soaked in the honey solution, placed into a 2-inch diameter petri dish, and placed in a cage for feeding. The cotton was replaced after 24 h. The culture was maintained under controlled conditions (25 ± 1 °C, 70 ± 5% RH, and 16:8 h (L:D) photoperiod), and experiments were conducted under identical conditions.

### 4.2. UV-Irradiation Exposure

During testing, *P. xylostella* was irradiated at 15 W by UV-C radiation (X-series, peak emission 254 nm; Spectronics, Westbury, NY, USA). All larvae were placed for two hours in darkness before UV-C exposure. Five treatments were established: 0 (control), 3 h, 6 h, 12 h, and 24 h UV-C exposure under dark conditions. Each treatment contained ten third instar *P. xylostella* larvae per replication, and three replications per treatment were established. After exposure to UV-C irradiation for different time durations, the insects were immediately frozen with liquid nitrogen and stored at −80 °C until the enzymatic activity was assessed.

### 4.3. Effect of UV-C Radiation on the Physiology of Plutella xylostella

#### 4.3.1. Sample Preparation

The five third instar larvae of *P. xylostella* were first weighed, and then samples were homogenized in an iced buffer (0.1 M phosphate buffer, 0.1 mM EDTA-2Na, 10 mM saccharose, 0.9% NaCl, pH = 7.4) with a bodyweight ratio of 0.1 g to 1 mL buffer. Next, homogenates were centrifuged for 20 min at 2500 RPM at 4 °C, and the supernatant was used for subsequent analyzes.

#### 4.3.2. Antioxidant Enzyme Activity Assay

ELISA kits (Jianglai Biotechnology Co., Ltd., Shanghai, China) were used to determine antioxidant enzyme activity. The double-antibody sandwiching system was adopted to determine the enzyme activity and related substance content. The activity of SOD, POD, PPO, CAT, and GST was assessed. Using SOD as an example, a microplate was covered with pure SOD antibody to form a solid phase antibody, and SOD was simultaneously applied to the coated monoclonal microcapsule. Then, SOD was combined with horseradish peroxidase (HRP)-labelled SOD antibody to form an antibody-antigen-enzyme-labelled antibody complex. After thorough washing, substrates 3, 3′, 5, 5′-tetramethylbenzidine (TMB) were used for color development. TMB was transformed to blue under HRP enzyme catalysis and converted to its final yellow color through acid activity. The depth of color in the test sample was positively correlated with SOD. The absorbance (OD value) was measured at a wavelength of 450 nm using an enzyme-labelling instrument (SpectraMax Plus 384, Molecular Devices Co., Ltd., Silicon Valley, CA, USA), and the sample concentration of SOD activity was determined from a standard curve.

#### 4.3.3. Detoxifying Enzyme Activity Assay

ELISA kits (Jianglai Biotechnology Co., Ltd., Shanghai, China) were used to test the key detoxifying enzymes, including ACP, CarE, ALP, and AChE, as per the procedures outlined in the user manual.

### 4.4. Effect of UV-C Radiation on Gene Regulation of Plutella xylostella

To determine the effect of UV-C radiation on gene regulation, two genes of each enzyme significantly correlated with UV-C radiation exposure time were selected and subjected to gene regulation quantification via qRT-PCR (Table 1).

#### 4.4.1. RNA Extraction and cDNA Synthesis

Total RNA was isolated using TRIzol Reagent (Invitrogen, Waltham, MA, USA); 1 mL TRIzol was added to a ground sample on ice and left open at room temperature for 5 min. Next, 200 μL chloroform/1 mL TRIzol was added and, after thorough shaking, left at room temperature for 15 min. The mixture was centrifuged at 4 °C at 12,000 rpm for 15 min. The upper aqueous phase was moved into a new centrifuge tube. Then, 0.5 mL isopropanol/1 mL TRIzol was added, and after shaking, left on ice for 10 min. The mixture was again centrifuged at 4 °C at 12,000 rpm for 10 min. The supernatant was removed from the tube, leaving only RNA pellets. Next, 75% ethanol 1 mL/1 mL of TRIzol was added to wash the pellet via centrifuging at 4 °C at 8000 rpm for 5 min. The washed liquid was discarded, and the RNA pellet was air-dried for 5–10 min. The RNA pellet was then re-suspended in RNase-free water (20–50 μL) and stored at –80 °C.

To prepare first-strand cDNA, 1 μg of total RNA was used. PrimeScript™ RT reagent Kit with gDNA Eraser (Perfect Real Time) (Takara, Japan) was used according to the manufacturer’s instructions. The cDNAs were diluted tenfold before the following quantitative real-time RT-PCR reactions (qRT-PCR).

#### 4.4.2. Primer Design and Testing

The gene sequences were obtained from NCBI, and the primers were designed using the CDS region. A 25 µL reaction mixture containing LA Taq DNA polymerase was used (Takara, Japan). The PCR conditions used in this study were previously explained by Guo et al. (2020). The pair of primers expressed as a single band was used in RT-qPCR.

#### 4.4.3. RT-qPCR with SYBR Green

A total 50 μL reaction volume contained 2.5 μL of each primer, 25 μL SYBR Premix (Takara, Japan), 2.5 μL diluted cDNA template, and 17.5 μL of RNase-free water. The above reaction solution was separated into three technical repeats containing 15 µL of the reaction mixture. All reactions were performed using the CFX96 real-time PCR system (Bio-Rad, Hercules, CA, USA). The qPCR program included an initial denaturation for 3 min at 95 °C followed by 40 cycles of 95 °C for 10 s and 55 °C for 30 s. Dissociation curve analysis was performed for each reaction to confirm the amplification specificity; a dissociation step cycle (55 °C for 10 s, and then 0.5 °C for 10 s until 95 °C) was added. RPS13 was used as the reference gene. The relative gene expression was computed using the 2-ΔΔCt method [84,85,86]. Details concerning the primers used are given in Table 1.

### 4.5. Preparation of the Conidial Suspension

*Cordyceps fumosorosea* fungal culture (Sp535) originally isolated from soil was obtained from the repository of the Key Laboratory of Biopesticides Innovation and Application of Guangdong Province, SCAU, and cultured on potato dextrose agar (PDA) in Petri dishes (9 cm in diameter) for ten days in an incubator at 25 ± 1 °C. Conidia were collected under sterile conditions in deionized water with 0.1% *v*/*v* Tween-80. The suspension was shaken for 20 min on a magnetized stirrer to break conidial clumps and then filtered to remove debris via four-layer medical gauze. The conidial concentration was determined and diluted up to 107 conidia/mL with a hemocytometer. Conidial viability was assessed by spraying 0.1 mL of the diluted spore suspension onto PDA and then testing the amount of germinating conidia at 25 °C after 24 h incubation [87,88]. In all tests, conidial suspension demonstrated viability of >95%.

### 4.6. Virulence Assessment Bioassay

Virulence of the fungi was assessed in two ways; (1) Larvae were treated with fungal suspension before exposure to UV-C radiation; (2) Larvae were treated with fungal suspension after UV-C radiation exposure. Four replications were set for five exposure times (0, 3, 6, 12, and 24 h). Each replicate contained fifteen third instar larvae of *P. xylostella* kept in a single Petri plate. Larvae were kept in 9 cm Ø Petri dishes with sufficient food (Chinese cabbage leaves).

For the bioassay of larvae treated with fungal suspension before UV-C exposure, the larvae were dipped into the conidial suspension for 5 s. They were then sieved out from suspension and kept at room temperature for 5 min to dry. The larvae were then transferred onto cabbage leaves in a Petri dish. The Petri dish was covered with 60 nylon mesh to prevent the larvae from escaping. Larvae were kept in the dark for 2 h before UV-C radiation exposure. Petri dishes were placed 50 cm below the UV-C radiation source. After 3, 6, 12, and 24 h, the Petri dishes were removed from the UV-C radiation source and placed in a climate-controlled chamber (25 ± 1 °C, 70–80% RH and 16:8 h (L:D) photoperiod). Mortality data were collected after 24, 48, and 72 h. The same methodology was adopted for other bioassays in which larvae were treated with *C. fumosorosea* before exposure to UV-C radiation.

To assess whether the UV-C radiation affected the virulence of *C. fumosorosea*, dead larvae from the bioassay in which fungal treatment was applied to larvae before UV-C radiation were kept on PDA plates. The grown fungi from larvae were recultured and subjected to the virulence test with the method outlined above.

### 4.7. Statistical Analysis

The corrected mortality (%) was calculated using Henderson-Tilton’s formula [89]. Variation of larval mortality among different fungal treatments, enzyme activity, and relative gene expression was computed using One-Way ANOVA, and significance among the mean treatment values was assessed with Tukey post hoc test at *P* < 0.05 using SPSS software. Replications were used to calculate the standard deviation of the mean. Mortality rate, enzymatic activity, and relative gene expression graphs were constructed by Sigmaplot 12.0. The Pearson correlation analysis was conducted to determine the relation between UV exposure time and the activity of different enzymes. The correlation matrix graph was designed with R software × 64 3.6.3 using the GGally package.

## 5. Conclusions

In conclusion, UV-C light works as an immunosuppressor by interfering with the antioxidant and detoxification enzyme activity, which leads to a reduction in the immunity of *P. xylostella* and an increase in virulence of the *C. fumosorosea*, if UV-C is applied to larvae before application of fungi. However, if UV-C irradiation is applied after the application of fungi, due to its germicidal effect, the virulence of *C. fumosorosea* could be reduced. This study is applicable under laboratory and greenhouse conditions, where synthetic pesticides cannot be used and quick and effective pest management is required. But before applying in the greenhouse, the effect of UV-C on plants must be studied. The molecular mechanism of an insect under UV stress after exposure to UV-C irradiation was found to interfere with the virulence of the entomopathogenic fungus. However, these effects were not permanent and were limited to a single generation. Furthermore, UV-light exposure disrupted antioxidant and detoxifying enzyme activity in *P. xylostella*; this change in enzyme activity may be due to altered gene expression in *P. xylostella*. This current study provides additional information regarding the efficacy of UV-C radiation against insect pests, and with further research, UV-C radiation may be employed as a pest management strategy.

## Figures and Tables

**Table 1:** The list of genes and primers of genes used on qRT-PCR.

Gene Name	NCBI Reference Sequence Number	Gene Name	Sequence (5′ to 3′)	Sequence
*CAT1*	XM_011560295.1	PREDICTED: *Plutella xylostella* catalase-like (LOC105389213), mRNA	F	ggctcaacgacaacctcatccg
			R	cgtgcgtgacctcgaagtagc
*CAT2*	XM_011561829.1	PREDICTED: *Plutella xylostella* catalase-like (LOC105390515), mRNA	F	caccaagtattccgccgccaag
			R	tccgcccaccgtcgagaatc
*CarE1*	XM_011552809.1	PREDICTED: *Plutella xylostella* carboxylesterase 1C-like (LOC105382842), mRNA	F	catgggaaagtatccgggaacacg
			R	tggttgtggtggcagaaatctcag
*CarE2*	XM_011558702.1	PREDICTED: *Plutella xylostella* carboxylesterase 1E-like (LOC105387900), mRNA	F	actgcccatgccaagaccaaac
			R	agacgctcgccttagctccag
*PPO1*	NW_011952494.1	*Plutella xylostella* strain DBM-FZ-S unplaced genomic scaffold, DBM_FJ_V1.1 scaffold_467, whole genome shotgun sequence	F	agccataggaagcctgacctcatc
			R	gctgacgacaccgaccacaat
*PPO2*	XM_011565232.1	PREDICTED: *Plutella xylostella* uncharacterized LOC105393465 (LOC105393465), mRNA	F	ggagtgaagccgccgaaagc
			R	tgttgccaccgataatccgatcag
*RPS13*	NM_001017.3	Ribosomal protein S13	F	tcaggcttattctcgtcg
			R	gctgtgctggattcgtac

## Data Availability

All the data are available in the manuscript file.
